# ^103^Pd versus ^125^I ophthalmic plaque brachytherapy: preoperative comparative radiation dosimetry for 319 uveal melanomas

**DOI:** 10.1007/s13566-014-0149-4

**Published:** 2014-04-09

**Authors:** Paul T. Finger, Di Zhou, Nina Kalach, Ekaterina Semenova, Walter Choi

**Affiliations:** 1 The New York Eye Cancer Center, 115 East 61st Street, New York City, NY 10065 USA; 2 Mt. Sinai Beth Israel Cancer Center, New York City, NY USA; 3 The New York Eye and Ear Infirmary of Mt. Sinai, New York City, NY USA; 4 New York University School of Medicine, New York City, NY USA

**Keywords:** Brachytherapy, Plaque, Palladium-103, Iodine-125, Choroidal melanoma

## Abstract

**Objective:**

This study was conducted to compare the relative, clinical intraocular dose distribution for palladium-103 (^103^Pd) versus iodine-125 (^125^I) ophthalmic plaque radiation therapy.

**Methods:**

Preoperative comparative radiation dosimetry was performed to evaluate 319 consecutive uveal melanomas treated between 2006 and 2012.

**Results:**

There were 68 (21.3 %) anterior (iris and/or ciliary body) and 251 (78.7 %) choroidal melanomas examined in this study. According to AJCC staging, 7th edition, 146 (45.8 %) were T1, 126 (39.5 %) T2, 40 (12.5 %) T3, and 7 (2.2 %) T4. All were prescribed an equivalent tumor-apex dose. When compared to ^125^I, ^103^Pd was associated with a mean 41.9 % lower radiation dose to the opposite eye wall (*p* < 0.001), 12.7 % to the lens center (*p* < 0.001), 7.5 % to the optic disc (*p* = 0.008), and a 3.8 % decrease to the fovea (*p* = 0.034). However, subgroup analysis of smaller (T1-staged) tumors showed greater dose reductions to normal ocular structures compared to larger (T4-staged) tumors. Tumor and therefore plaque location also affected intraocular dose distribution. For example, palladium-103-related dose reductions to the fovea, optic nerve, and opposite eye wall were significantly greater for iris and ciliary body tumors compared to posterior choroidal melanomas (*p* < 0.001). After comparative dosimetry, 98.7 % (*n* = 315/319) were treated with ^103^Pd.

**Conclusion:**

Preoperative comparative radiation dosimetry was performed for a large cohort of patients with uveal melanoma. It influenced radionuclide selection, offered an opportunity for radiation sparing of critical vision-related intraocular structures, and typically increased radiation within the tumors.

## Introduction

Complications of plaque brachytherapy have been related to dose to normal ocular structures [1–5]. However, little has been written about methods to diminish unnecessary irradiation outside the targeted treatment volume [6–10, 5, 11].

In 1990, the first preclinical pilot studies used computer simulations and then thin layer dosimeter (TLD)-laden frozen eye bank eyes to compare ^103^Pd and ^125^I ocular dose distributions [8]. For an equivalent tumor target dose, the less energetic (21 keV) ^103^Pd-photons were more quickly absorbed than those from the (28 keV) ^125^I plaques within the eye and vitreous before it reached the episcleral dosimeters. In clinical practice, it was reasonable to assume that when outside the targeted volume (beneath the plaque), there would be relative ^103^Pd-related dose reductions to the macula, optic nerve, and lens. In 2009, the clinical results of 400 cases of choroidal melanoma treated by ^103^Pd ophthalmic plaque therapy demonstrated improved local control and visual acuity outcomes compared to other series using ^125^I [12].

In 2011, the American Association of Physicists in Medicine (AAPM) Task Group-129 (TG-129) examined the relative ocular dose distributions of ^125^I versus ^103^Pd plaque treatment for a single moderately sized T1 tumor and found relative dose advantages with the use of ^103^Pd [11]. In a second publication together with the American Brachytherapy Society (ABS), the AAPM TG-129 suggested that comparative preoperative dosimetry could improve clinical care [13]. Most recently, the ABS Ophthalmic Oncology Task Force guidelines reached consensus that a pretreatment comparison of available sources be employed prior to plaque therapy [14].

Since 2006, at The New York Eye Cancer Center and affiliated hospitals, we have routinely compared ^125^I to ^103^Pd prior to ophthalmic plaque brachytherapy. Utilizing data from those evaluations, we present the largest clinical case series of preoperative intraocular dosimetric comparisons for ophthalmic plaque brachytherapy for uveal melanoma.

## Methods

### Clinical evaluation and diagnosis

Preoperative comparative (^103^Pd versus ^125^I) dosimetry calculations were performed for 319 uveal melanoma patients treated between 2006 and 2012. Patients were diagnosed with uveal melanoma by clinical examination. A combination of ophthalmoscopy, fluorescein angiography, transillumination, and B-scan ultrasound were used to determine the tumor’s basal dimensions and apical height. Anterior uveal tumors (defined as iris and ciliary body melanomas) were assessed by slit-lamp biomicroscopy, gonioscopy, high-frequency ultrasound imaging and photography. Our methods of diagnosis are consistent with that described in the ABS/AAPM TG-129 report [13]. All tumors were T-staged according to the American Joint Committee on Cancer (AJCC) System, 7th edition [15–18].

### Dosimetric calculations and treatment

Preoperative dosimetry calculations for ^103^Pd and ^125^I were performed to be comparable to the COMS protocol and followed the current recommendations of the American Association of Physicists in Medicine Task Group-43 (AAPM TG-43) [19, 13, 11, 20–22]. Thus, seeds were calculated as approximate isotropic point sources with no corrections for anisotropy. No attenuation was attributed to the acrylic fixative or for the gold plaque sidewalls. The backscatter effects from the plaque’s posterior wall were discounted. A ^103^Pd specific dose rate constant of 0.686 cGy/h/mCi was used. The radial dose function was obtained from published data [23–25]. The prescription point was the tumor’s apex, consistent with the 2003 and most current ABS recommendations [22, 14].

We chose to analyze four points along the central axis of the plaque: inner sclera, 5-mm axial depth, opposite retina, and the tumor’s apex. Due to their importance for visual acuity, additional intraocular locations included: the fovea, optic disc, and lens.

### Statistical analysis

Comparison of the ^103^Pd and ^125^I ophthalmic plaque doses was computed using the Mann–Whitney *U* test. A confidence interval (CI) of 95 % was generated from the statistical analysis. In comparison of ^103^Pd and ^125^I ophthalmic plaque doses, a *P* value < 0.05 was considered statistically significant.

## Results

### Tumor characteristics

There were 319 uveal melanomas evaluated by comparative dosimetry between 2006 and 2012. They had a mean 4.0 mm apical height (range 1.8–14.2) and a mean 9.9 mm basal dimension (range 1.8–21). The majority were AJCC T1 and T2 stages at 45.8 % and 39.5 %, respectively (Table 1). This is comprised of iris or ciliary body (*n* = 68/319, 21.3 %), but majority were choroidal melanomas (*n* = 251/319, 78.7 %).

**Table 1:** Melanoma characteristics and location

		Percentages (*n*)^*^
Tumor stage	T1	45.8 % (146)
	T2	39.5 % (126)
	T3	12.5 % (40)
	T4	2.2 % (7)
Tumor location	Anterior (iris and ciliary body)	21.3 % (68)
	Choroid	78.7 % (251)

*n* number of patients

### Comparative dosimetry

#### Central axis dose

**Fig. 1:**
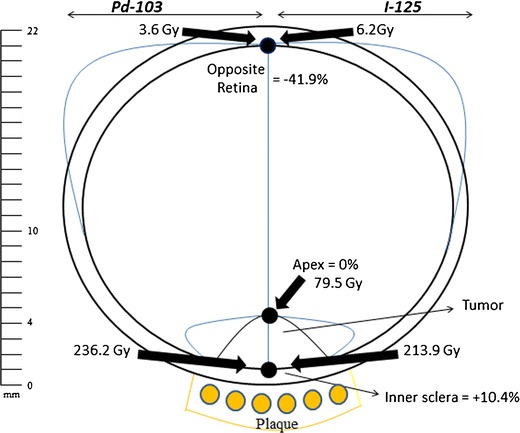
Dose gradient along the central axis of the plaque

All treatment plans were analyzed for the relative dose deposition along the central axis of the plaque (inner sclera, 5 mm and opposite eye wall). By definition, the tumor’s apex received a mean equivalent dose for both radionuclides (Fig. 1). However, the use of ^103^Pd (versus ^125^I) was associated with a mean 10.4 % increase in dose to the inner scleral base (*p* = 0.040) and a 1.1 % increase at 5-mm axial depth (*p* = 0.031) for that equivalent tumor-apex dose. Therefore, the use of ^103^Pd was found to steepen the dose gradient within the tumor. Specifically, for an equivalent tumor-apex dose, the use of ^103^Pd increased the mean radiation dose within the tumor by mean 7.6 % (157.9 versus 146.7 Gy) compared to ^125^I.

Then as the lower energy ^103^Pd photons continue along the central axis across the eye, they were more rapidly absorbed (in the vitreous) before it reached the opposite eye wall (Fig. 1). At that data point, the use of ^103^Pd resulted in a 41.9 % reduction of radiation dose (*p* < 0.001).

#### Dose to critical intraocular structures

Vision retention is related to the function of certain critical radiosensitive intraocular structures. Therefore, we compared the radiation dose to the natural lens center, optic nerve, and fovea. Depending on the intraocular location of the tumor (and therefore the plaque), these critical structures can be located either within or outside the target volume [26]. In this series, the use of ^103^Pd (versus ^125^I) demonstrated overall mean reductions to these critical intraocular structures (Table 2). Specifically, the optic disc and fovea showed a mean ^103^Pd-related reduction of 7.5 % (*p* = 0.008) and 3.8 % (*p* = 0.034), respectively. The lens center showed a greater mean ^103^Pd-related radiation dose reduction of 12.7 % (*p* < 0.001).

**Table 2:** Preoperative dosimetry for all melanomas, melanomas of the iris and ciliary body, and posterior choroidal melanomas

	All tumors ^103^Pd: mean dose, Gy	All tumors ^125^I: mean dose, Gy	Mean absolute dose difference between ^103^Pd and ^125^I, Gy	Mean ^103^Pd change relative to mean ^125^I dose, percent	*P* value	Anterior tumors ^103^Pd: mean dose, Gy	Anterior tumors ^125^I: mean dose, Gy	Mean absolute dose difference between ^103^Pd and ^125^I, Gy	Mean ^103^Pd change relative to mean ^125^I dose, percent	*P* value	Posterior Choroidal tumors ^103^Pd: mean dose, Gy	Posterior Choroidal tumors ^125^I: mean dose, Gy	Mean absolute dose difference between ^103^Pd and ^125^I, Gy	Mean ^103^Pd change relative to mean ^125^I dose, %	*P* value
	*N* = 319	*N* = 319	*N* = 319	*N* = 319		*N* = 68	*N* = 68	*N* = 68	*N* = 68		*N* = 251	*N* = 251	*N* = 251	*N* = 251	
Inner sclera (beneath the plaque)	236.2, CI 222.1–250.3	213.9, CI 204.0–223.8	22.3	+10.4 %	0.040	234.5, CI 196.6–272.4	215.2, CI 188.6–241.7	19.3	+9.0 %	0.575	236.7, CI 221.9–251.5	213.6, CI 203.1–224.1	27.1	+10.8 %	0.062
5-mm Depth	64.8, CI 60.0–70.0	64.1, CI 60.3–67.9	0.7	+1.1 %	0.031	59.3, CI 45.6–72.3	58.9, CI 48.2–69.3	0.4	+0.7 %	0.024	66.4, CI 61.4–71.3	65.6, CI 61.6–69.5	0.8	+1.2 %	0.101
Prescription point (apex of tumor)	79.5, CI 78.6–80.4	79.5, CI 78.7–80.4	0.0	0.0 %	1.000	81.2, CI 79.3–83.1	81.2, CI 79.3–83.1	0.0	0.0 %	0.998	79.0, CI 78.0–80.0	79.0, CI 78.0–80.0	0.0	0.0 %	1.000
Opposite retina	3.6, CI 3.0–4.2	6.2, CI 5.4–7.0	2.6	−41.9 %	<0.001	3.1, CI 2.0–4.3	5.3, CI 3.9–6.7	2.2	−41.5 %	<0.001	3.7, CI 3.0–4.4	6.5, CI 5.5–7.4	2.8	−43.1 %	<0.001
Lens center	20.0, CI 17.5–22.4	22.9, CI 20.6–25.1	2.9	−12.7 %	<0.001	42.9, CI 37.6–48.1	45.0, CI 40.2–49.8	2.1	−4.7 %	0.285	13.7, CI 11.5–15.9	17.8, CI 14.7–21.0	4.1	−23.0 %	<0.001
Optic disc	28.4, CI 24.1–32.7	30.7, CI 26.7–34.6	2.3	−7.5 %	0.008	6.8, CI 1.4–12.3	8.5, CI 3.9–13.0	1.7	−20.0 %	<0.001	34.3, CI 29.2–39.3	36.7, CI 32.1–41.3	2.4	−6.5 %	0.040
Macula (fovea)	37.9, CI 33.0–42.8	39.4, CI 34.8–44.0	1.5	−3.8 %	0.034	7.4, CI 1.4–13.4	8.9, CI 3.8–14.0	1.5	−16.9 %	<0.001	46.2, CI 40.5–51.8	47.7, CI 42.5–52.9	1.5	−3.1 %	0.175

*Gy* Gray, *CI* 95 % confidence interval

Dose is given in Gray (1 Gy = 100 cGy = 100 Rad)

### Comparing anterior versus posterior tumor location

The subgroups of 68 anterior (iris and ciliary body) and 251 choroidal melanomas had relatively equivalent mean apical heights (3.8 versus 4.1 mm, respectively). Thus, both groups generally resulted in ^103^Pd-related increased radiation dose to the subjacent inner sclera and to the 5-mm axial depth (Fig. 1). Similarly, the use of ^103^Pd reduced the radiation dose to the opposite retina by 41.5 % (*p* < 0.001) and 43.1 % (*p* < 0.001) for anterior and posterior melanomas, respectively.

**Fig. 2:**
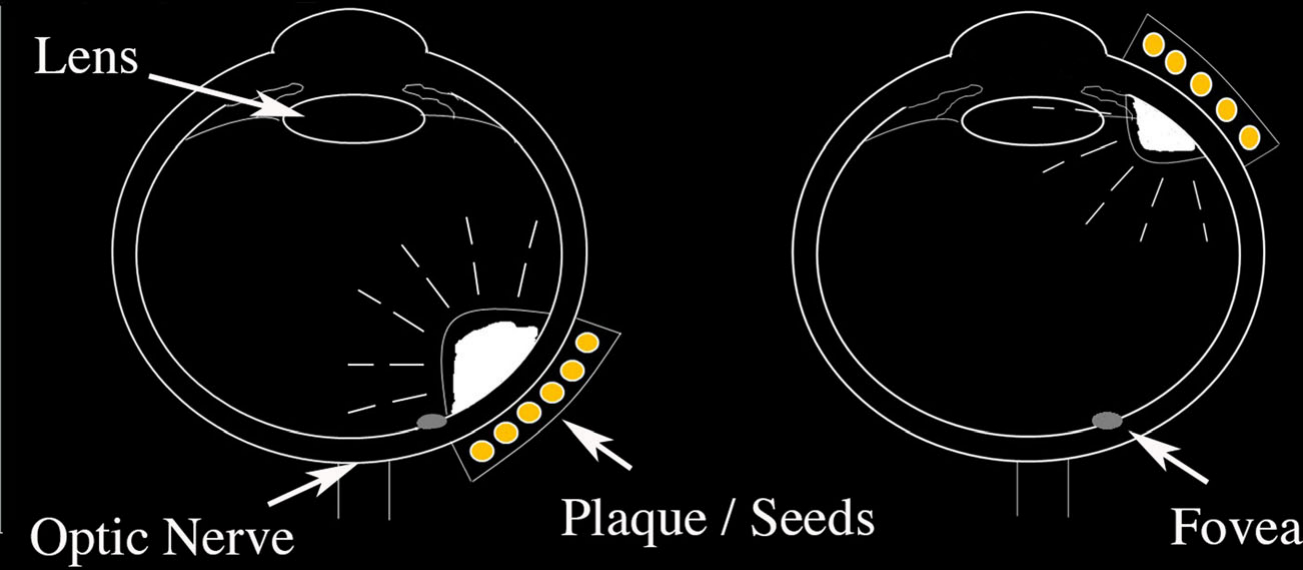
Tumor location affects distance to critical normal ocular structures

However, this subgroup analysis (anterior versus posteriorly located tumors) also revealed that the tumors’ relative distance to the lens, fovea, and optic disc was markedly different (Fig. 2). Specifically, anterior tumors were in closer proximity to the natural lens and farther from the macular fovea and optic disc. Conversely, posterior tumors were in closer proximity to the fovea and optic disc. These differences were found to affect the relative dose distributions for ^103^Pd versus ^125^I plaques.

Specifically, in treatment of anterior tumors, ^103^Pd offered relative dose reductions of a mean 4.7 % (*p* = 0.285) to the relatively close lens. However, in treatment of posteriorly located melanomas, the use of ^103^Pd reduced the lens dose by 23.0 % (*p* < 0.001). Similarly, in evaluation of the fovea and optic disc, the subset of anteriorly located tumors revealed mean ^103^Pd-related dose reductions of 16.9 % (*p* < 0.001) to the fovea and 20.0 % (*p* < 0.001) to the optic disc. However, by comparative dosimetry for the subset of posteriorly located choroidal melanomas, mean^103^Pd-related dose reductions were 3.1 % (*p* = 0.175) to the fovea and 6.5 % (*p* = 0.040) to the optic nerve. This analysis revealed that ^103^Pd typically reduced the mean dose to the lens, fovea, and optic disc. However, the magnitude of that difference was dependent on distance.

### AJCC T stage tumor comparison

**Fig. 3:**
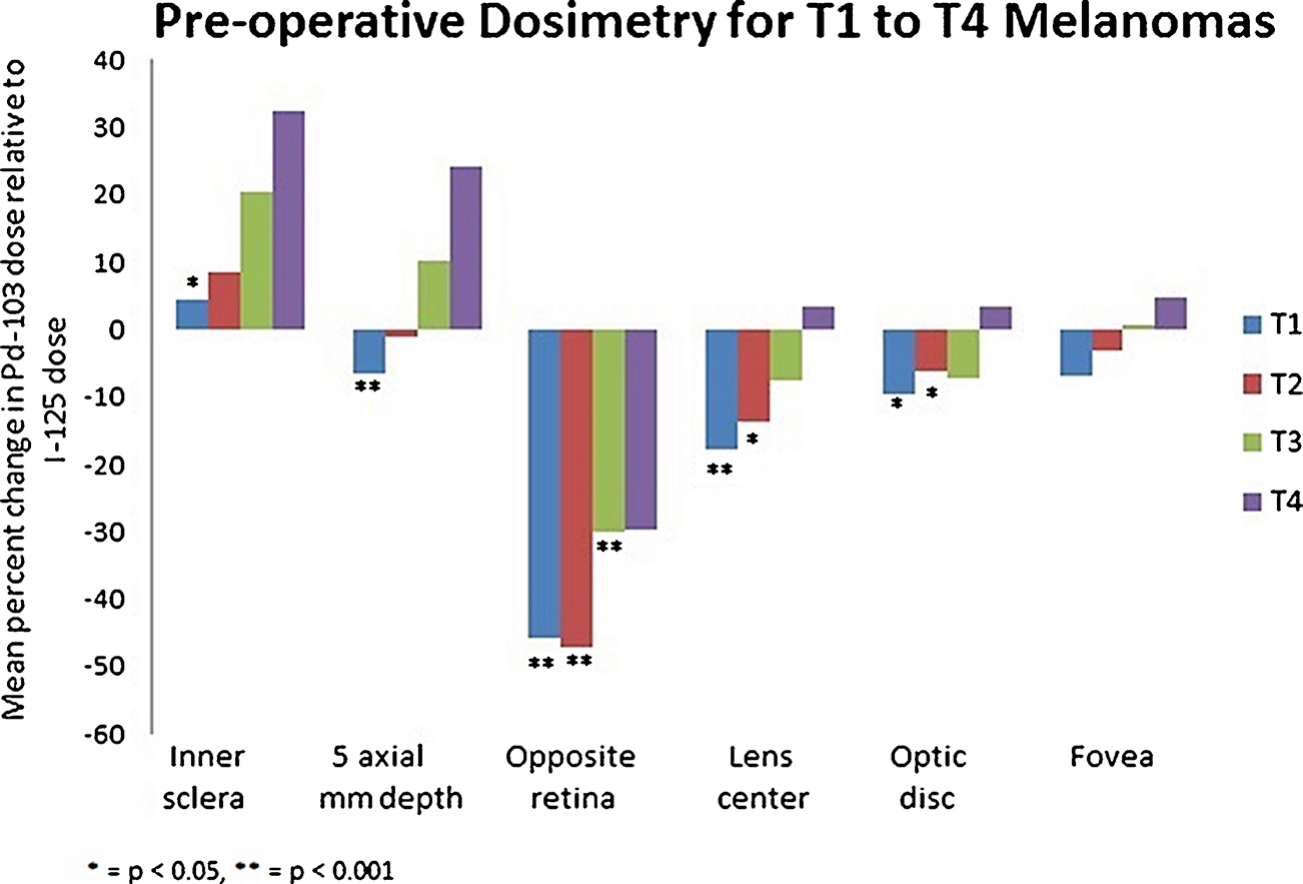
Mean percent change in dose when comparing ^103^Pd versus ^125^I

Dosimetry of ^103^Pd versus ^125^I was also analyzed by AJCC T staging (Table 3). The most significant differences were noted between the relatively small T1 and the large T4 melanomas. For example, axial dosimetry for T1-staged tumors (*n* = 146) and T4-staged tumors (*n* = 7) revealed that ^103^Pd was associated with a mean increase in dose to the inner sclera of 4.5 % (*p* = 0.066) versus 32.2 % (*p* = 0.208), respectively. This shows that while all tumors received a higher mean scleral dose using ^103^Pd, the dose difference increased with tumor size. Similarly, the radiation dose to a 5-mm axial depth demonstrated a decrease of 6.6 % with ^103^Pd for the relatively short T1 tumors and an increase of 24.2 % for T4 tumors (Fig. 3).

**Table 3:** Preoperative dosimetry for stages T1–T4 melanomas

	T1 tumors ^103^Pd: mean dose, Gy	T1 tumors ^125^I: mean dose, Gy	Mean absolute dose difference between ^103^Pd and ^125^I, Gy	Mean ^103^Pd change relative to mean ^125^I dose, percent	*P* value	T2 tumors ^103^Pd: mean dose, Gy	T2 tumors ^125^I: mean dose, Gy	Mean absolute dose difference between ^103^Pd and ^125^I, Gy	Mean ^103^Pd change relative to mean ^125^I dose, percent	*P* value
	*N* = 146	*N* = 146	*N* = 146	*N* = 146		*N* = 126	*N* = 126	*N* = 126	*N* = 126	
Inner sclera (beneath the plaque)	177.7, CI 156.9–198.5	170.0, CI 155.4–184.6	7.7	+4.5 %	0.066	233.5, CI 218.0–249.1	215.4, CI 203.8–227.0	18.1	+8.4 %	0.131
5-mm depth	42.5, CI 35.3–49.7	45.5, CI 39.8–51.2	3.0	−6.6 %	<0.001	65.4, CI 60.4–70.3	66.2, CI 61.8–70.5	0.8	−1.2 %	0.516
Prescription point (apex of tumor)	82.4, CI 81.1–83.7	82.4, CI 81.1–83.7	0.0	0.0 %	0.999	79.8, CI 78.7–80.8	79.8, CI 78.7–80.8	0.0	0.0 %	0.999
Opposite retina	1.9, CI 1.0–2.8	3.7, CI 2.5–4.9	1.7	−45.9 %	<0.001	3.6, CI 2.9–4.3	6.8, CI 5.3–8.2	3.2	−47.1 %	<0.001
Lens center	12.9, CI 9.3–16.5	15.7, CI 12.4–19.0	2.8	−17.8 %	<0.001	21.8, CI 18.4–25.3	25.3, CI 22.0–28.6	3.5	−13.8 %	0.001
Optic disc	23.9, CI 17.5–30.3	26.4, CI 20.5–32.3	2.5	−9.5 %	0.039	29.6, CI 22.0–37.2	31.6, CI 24.6–38.6	2.0	−6.3 %	0.064
Macula (fovea)	32,8, CI 25.6–40.0	35.2, CI 28.4–42.0	2.4	−6.8 %	0.091	37.7, CI 29.8–45.6	39.0, CI 31.6–46.4	1.3	−3.3 %	0.142
	T3 tumors ^103^Pd: mean dose, Gy	T3 tumors ^125^I: mean dose, Gy	Mean absolute dose difference between ^103^Pd and ^125^I, Gy	Mean ^103^Pd change relative to mean ^125^I dose, percent	*P* value	T4 tumors ^103^Pd: mean dose, Gy	T4 tumors ^125^I: mean dose, Gy	Mean absolute dose difference between ^103^Pd and ^125^I, Gy	Mean ^103^Pd change relative to mean ^125^I dose, percent	*P* value
	*N* = 40	*N* = 40	*N* = 40	*N* = 40		*N* = 7	*N* = 7	*N* = 7	*N* = 7	
Inner sclera (beneath the plaque)	386.3, CI 337.7–434.8	321.2, CI 289.3–353.2	65.1	+20.3 %	0.051	647.8, CI 394.8–900.9	490.0, CI 330.5–649.5	157.8	+32.2 %	0.208
5-mm Depth	118.5, CI 101.4–135.6	107.4, CI 94.3–120.5	11.1	+10.3 %	0.308	210.3, CI 127.9–292.8	169.3, CI 114.4–224.2	41.0	+24.2 %	0.401
Prescription point (apex of tumor)	70.8, CI 67.4–74.2	70.8, CI 67.4–74.2	0.0	0.0 %	0.996	62.6, CI 51.0–74.3	62.6, CI 51.0–74.3	0.0	0.0 %	0.952
Opposite retina	8.4, CI 4.5–12.5	12.0, CI 8.2–15.9	3.6	−30.0 %	<0.001	11.5, CI 7.0–16.0	16.4, CI 11.1–21.7	4.9	−29.9 %	0.208
Lens center	31.9, CI 21.4–42.4	34.5, CI 25.5–43.5	2.6	−7.5 %	0.159	50.9, CI −1.7–103.5	49.2, CI 8.4–90.1	1.7	+3.5 %	0.998
Optic disc	37.3, CI 27.5–47.1	40.2, CI 30.9–49.5	2.9	−7.2 %	0.407	65.2, CI 35.1–95.3	63.2, CI 40.5–86.0	2.0	+3.2 %	0.912
Macula (fovea)	54.0, CI 37.6–70.3	53.6, CI 39.8–67.5	0.4	+0.7 %	0.582	56.6, CI −2.2–115.4	54.0, CI 7.6–100.4	2.6	+4.8 %	0.910

Dose is given in Gy = Gray (1 Gy = 100 cGy = 100 Rad), CI = 95 % confidence interval

Once the radiation extended beyond the equivalent tumor-apex prescription dose, ^103^Pd radiation was absorbed by the vitreous before reaching the opposite eye wall. Dose to this data point was also affected by tumor height. For example, in treatment of taller tumors, there was less intervening vitreous or distance to the opposite eye wall. Thus, ^103^Pd-related mean dose reductions to the opposite eye wall were 45.9 % (*p* < 0.001) for T1 tumors versus 29.9 % (*p* = 0.208) for T4 tumors (Table 3).

However, all the important anatomical ocular structures (lens center, optic disc, and fovea) demonstrated mean dose reduction with ^103^Pd versus ^125^I for T1 to T3 tumors (Fig. 3). Here, comparative dosimetry revealed that the use of ^103^Pd decreased the mean dose to the opposite eye wall, while ^103^Pd increased the mean dose to the lens, optic disc, and fovea in T4-staged uveal melanomas (Fig. 3). Detailed subset analysis demonstrated that the use of ^103^Pd would increase the dose (for T4-staged tumors) to the lens and fovea in three eyes (*n* = 3/7, 43 %) and to the optic disc in two eyes (*n* = 2/7, 29 %). No eye with a tumor in the T4 subset exhibited a relative increase to all three ocular structures (optic disc, fovea, and lens) using ^103^Pd.

### Patients treated with ^125^I plaques

There were 315 patients (98.7 %) who received ^103^Pd plaque therapy and four patients who received ^125^I plaque therapy. The four tumors treated with ^125^I had a mean 11.7 mm apical height (range 8.2 to 14.2) and a mean 16.1 mm basal dimension (range 13.9–17.9). The distance from the tumor edge to the macula and optic disk ranged from 0 to 3.4 mm and from 0 to 3.9 mm, respectively. Of these four tumors, two were T3 and two were T4 tumors. In those four ^125^I-treated eyes, the lens received an increased ^125^I dose of 12.6 % relative to that of ^103^Pd. However, it was the mean ^125^I-related dose reduction at the optic disc and fovea (10.5 and 16.0 %, respectively) that was the main reason for using ^125^I. Overall, three patients received lower optic disc dose due to the use of ^125^I and all four patients received lower fovea dose with ^125^I.

## Discussion

Comparative clinical dosimetry was used to analyze intraocular dose distribution prior to radiation therapy for 319 uveal melanomas. Specifically, lower energy ^103^Pd was compared to relatively higher energy ^125^I seeds for ophthalmic plaque therapy. Evaluations along the central axis of the plaque revealed that the scleral dose was higher utilizing ^103^Pd. Therefore, with an equivalent planned apex prescription, the use of ^103^Pd increased the mean tumor dose. In addition, the opposite eye wall was both the farthest from the plaque and best represented organ dose. Here, the use of ^103^Pd was associated with an overall reduction of 41.9 % (*p* < 0.001). In comparison of critical targets for vision preservation, there were modest overall ^103^Pd-related dose reductions to the lens, optic disc, and fovea. While subset analysis of anteriorly located uveal melanomas revealed significantly greater ^103^Pd-related sparing of the optic disc and fovea, dosimetry for the posterior choroidal melanomas revealed more significant mean dose reductions to the lens.

Tumor size also influenced the intraocular dose distribution. In comparison of ^103^Pd versus ^125^I, increased T staging as per AJCC, 7th edition, was associated with relatively more tumor irradiation with ^103^Pd. For example, from T1 to T4, the mean percent increase in dose to the inner sclera rose from 4.5 % (*p* = 0.066), 8.4 % (*p* = 0.131), 20.3 % (*p* = 0.051), and 32.2 % (*p* = 0.208), respectively. Conversely, the mean percent decrease in dose to the opposite retina provided by ^103^Pd relative to ^125^I trended to diminish as the tumors get larger, T1 45.92 % (*p* < 0.001), T2 47.1 % (*p* < 0.001), T3 30.0 % (*p* < 0.001), and T4 29.9 % (*p* = 0.208). As their edges were more likely to be in close proximity to the lens center, optic disc, and fovea, higher T-staged (larger uveal melanomas) exhibited smaller percent differences between radionuclides. In these cases, it was not uncommon to find the calculated dose to these critical ocular structures beyond published tolerances. In addition, our decisions to use ^103^Pd were related to dose savings to the opposite eye wall in an effort to diminish organ dose.

The majority of tumors were treated with ^103^Pd, but 1.3 % tumors (*n* = 4/319) received ^125^I. In general, the decision to use ^125^I was influenced by the higher mean ^103^Pd dose at the optic disc (10.5 %) and fovea (16.0 %). The increased ^103^Pd dose at the optic disc and fovea was affected by the large tumor apical height and proximity of the optic disc and fovea to the tumor edge.

The limitations of this study include the following: it is from a single center, it was retrospective, and there was a small sample size of T4 tumors. Furthermore, the dosimetry calculations in this series assumed homogeneous water-equivalent tissues consistent with TG-43. The 2012 AAPM and ABS TG-129 recommended computing both homogenous and heterogeneous dose calculations as part of the preoperative plaque planning [13]. They noted that there can be large variations in doses between the two methods and that plaques often have heterogeneous material within that can affect dose calculations to normal intraocular structures [13]. Dosimetric calculations in this study also neglected anisotropy correction recommended by TG-43 to be in agreement with COMS practice [21, 23, 11]. As there are no universally accepted clinical dosimetry methods used at multiple ophthalmic brachytherapy centers, we present our findings as relatively equivalent to standard clinical practice during our period of recruitment. Though we recognize that different dosimetry methods may change the absolute numbers, there is no reason to believe they would change the dose distribution trends revealed by this work.

## Conclusion

This study demonstrates that pretreatment comparative dosimetry can be used to evaluate the intraocular distribution of radiation and aid radionuclide selection. Our study revealed that when comparing ^125^I versus ^103^Pd plaque therapy for uveal melanoma, both tumor size and location affected the relative dose to critical normal intraocular structures. In 319 patients, the use of ^103^Pd resulted in a trend toward increased dose within the tumor target volume and decreased dose to most normal ocular structures.
